# Femtosecond-Pulsed Laser Written and Etched Fiber Bragg Gratings for Fiber-Optical Biosensing

**DOI:** 10.3390/s18092844

**Published:** 2018-08-28

**Authors:** Sven Schulze, Michel Wehrhold, Carsten Hille

**Affiliations:** 1 Department of Physical Chemistry/Applied Laser Sensing in Complex Biosystems (ALS ComBi), University of Potsdam, 14476 Potsdam, Germany; sveschul@uni-potsdam.de (S.S.); Michel.Wehrhold@hu-berlin.de (M.W.); 2 School of Analytical Sciences Adlershof (SALSA), Humboldt-Universität zu Berlin, 10099 Berlin, Germany; 3 Current address: Technical University of Applied Sciences Wildau, 15745 Wildau, Germany

**Keywords:** fiber Bragg gratings, ultra-fast laser inscription, fiber etching, nanostructure fabrication, fiber-optical sensors, aptamers, C-reactive protein, biomarker

## Abstract

We present the development of a label-free, highly sensitive fiber-optical biosensor for online detection and quantification of biomolecules. Here, the advantages of etched fiber Bragg gratings (eFBG) were used, since they induce a narrowband Bragg wavelength peak in the reflection operation mode. The gratings were fabricated point-by-point via a nonlinear absorption process of a highly focused femtosecond-pulsed laser, without the need of prior coating removal or specific fiber doping. The sensitivity of the Bragg wavelength peak to the surrounding refractive index (SRI), as needed for biochemical sensing, was realized by fiber cladding removal using hydrofluoric acid etching. For evaluation of biosensing capabilities, eFBG fibers were biofunctionalized with a single-stranded DNA aptamer specific for binding the C-reactive protein (CRP). Thus, the CRP-sensitive eFBG fiber-optical biosensor showed a very low limit of detection of 0.82 pg/L, with a dynamic range of CRP detection from approximately 0.8 pg/L to 1.2 µg/L. The biosensor showed a high specificity to CRP even in the presence of interfering substances. These results suggest that the proposed biosensor is capable for quantification of CRP from trace amounts of clinical samples. In addition, the adaption of this eFBG fiber-optical biosensor for detection of other relevant analytes can be easily realized.

## 1. Introduction

Biomarkers as measurable and quantifiable biological parameters are definitely beneficial in diagnostic and therapeutic strategies, because they can indicate a variety of health and disease characteristics [1]. Thus, the detection and quantification of biomarkers is highly important nowadays [2,3,4] and there is still a high need of adequate detection methods. Biosensors are devices that can be used for effective and confident biomarker detection. They consist of a biological recognition unit element specifically interacting with the biological parameter target analyte and a transducer converting the biological signal to an electrical output [5,6]. Among others, optical biosensors exhibit beneficial properties, such as immunity to electromagnetic interferences, capabilities for real time and remote sensing, as well as multiplexing concepts [7].

Optical biosensors based on fiber Bragg gratings (FBG) exhibit unique features making them a favorable platform for biosensing [8,9]. A Bragg grating is a periodic modulation of the refractive index (RI) of the fiber core in longitudinal direction, creating an optical filter within the fiber [10]. The fiber-guided light is changed at the FBG from a forward propagating mode to a backward mode. Therefore, at the FBG, a narrow spectral band is reflected and the remaining light is transmitted. The wavelength of the reflected light is defined by the Bragg resonance wavelength (*λ*_B_). It depends on the harmonic order of the reflection (*m*), the grating period of the modulation (Λ), and the effective refractive index of the FBG region core mode (*n*_eff_), according to Ref. [11]:

(1) $$m\lambda_{B}=2\Lambda n_{\mathrm{eff}}$$

Several FBG fabrication methods have been developed so far [12]. The most widely used fabrication method is the phase mask technique [13]. For this, the phase mask grating (Λ_pm_) has a twofold grating period in comparison to the desired FBG grating (Λ_FBG_). When ultraviolet (UV) laser light is focused through a phase mask, the generated interference pattern of the first diffraction order of the phase mask can be then inscribed into the fiber core. However, this inscription process needs a high fiber photosensitivity. The highest photosensitivity can be obtained by germanium-doped fibers. Other post-fabrication techniques such as hydrogen loading, flame brushing, or boron co-doping can also increase the photosensitivity, however, for some techniques it works only transiently [12,14,15,16]. A further limitation of the phase mask technique is that only a single Bragg grating period can be obtained from a given phase mask and more complex grating structures would require more complex, expensive phase masks.

In the present study, FBGs were fabricated by an alternative method, the so-called point-by-point (PBP) technique or ultrafast-laser-inscription (ULI) [17,18,19]. Here, the focused femtosecond (*fs*)-pulsed laser interacts with the glass via nonlinear photoionization mechanisms, so that there is no need for glass photosensitivity or removing of fiber cladding/coating. Each laser pulse induces local RI changes in the fiber core and creates a grating pitch in the fiber [17]. The Bragg resonance wavelength of the resulting FBG (*λ*_B_) depends on the translation speed of the fiber (*v*) during the inscription process and the repetition rate of the *fs*-laser (*f*_rep_), and together with Equation (1) it can be calculated:

(2) $$\lambda_{B}=\frac{2n_{\mathrm{eff}}v}{mf_{\mathrm{rep}}}$$

In contrast to FBGs fabricated *via* phase mask technique, it is possible to fabricate FBGs with higher harmonic orders (*m* ≥ 2) by using ULI with changed ratio of translation speed and repetition rate [17]. Thus, one advantage of the PBP technique is its flexibility in creating very different gratings.

The sensing principle of FBG-based sensors is the measurement of the shift in the Bragg wavelength (Δ*λ*_B_), for which two different sensor concepts have been established. On the one hand, Δ*λ*_B_ can be observed due to changes in Λ as a result of altered external physical properties such as temperature [20] or straining [21,22,23] effects. Interestingly, FBGs written with PBP technique exhibit an enhanced thermal stability and are particularly suitable for temperature sensing [24]. On the other hand, Δ*λ*_B_ can be observed due to changes in *n*_eff_ as a result of an interaction of the guided light and the surrounding medium. In the latter case, the sensitivity of the FBG-sensor depends on the cladding thickness, because only part of the guided light propagates as evanescent wave into the cladding [25]. For removing the fiber cladding, different approaches have been reported in the past, such as side-polishing [26,27] or fiber etching with hydrofluoric acid (HF) [28,29]. Finally, the realized fiber diameter determines the degree of Δ*λ*_B_ as a result of an altered surrounding refractive index (SRI) [25]. However, the cladding removal is not the only way to achieve a sufficient sensitivity to SRI. The FBG technique can be also combined with microstructured optical fibers [30], hollow-core fibers [31], suspended-core fibers [32,33], or surface plasmon resonance (SPR) as plasmonic optical fiber [34]. Tilted fiber Bragg gratings (TFBG) are based on periodic modulations weakly tilted relative to the fiber axis. This leads to the coupling between the core mode and selected cladding modes, which are SRI sensitive and lead to a series of narrow spectral bands in the transmission spectrum. In the case of long period gratings (LPG), the coupling of core and cladding modes is achieved by increasing the grating period in the range of 100–1000 µm. This leads only to a coupling of the fundamental mode in the fiber core with co-propagating cladding modes [9]. Both, TFBG- and LPG-based sensors are sensitive to SRI without any changes in the fiber geometry making them favorable for several biosensor designs. Thus, biosensors are reported with biofunctionalization via cross-linking to a silane-functionalized fiber surface [35,36], coupling by using a layer-by-layer method [37], or *via* polymeric deposition [38,39]. However, SRI sensitivity can usually only be recorded in the transmission spectrum, when using TFBGs or LPGs [40]. Interestingly, a SPR-TFBG biosensor technology working in the reflection measurement mode has been reported recently [41]. The reflection measurement mode simplifies the recording setup having all hardware at one end of the fiber and can then allow for remote sensing also combined with reduced required sample volumes.

In this study, we present for the first time the realization of a fiber-optical biosensor, which combines PBP-written and etched FBGs (eFBG) fibers with a specific, single-stranded DNA aptamer as recognition element for biomarker detection. The FBGs were written into single-mode fibers by PBP-technique with high reproducibility. Here, the Δ*λ*_B_ sensitivity to SRI was significantly increased by chemical HF etching. The proof of principle of the realized biosensor was demonstrated using the well-known human biomarker C-reactive protein (CRP) [42]. CRP is an acute-phase protein in the blood plasma and its blood concentration level rises up to two orders of magnitude in the case of inflammation from approximately 5 mg/L up to 200 mg/L [43,44]. CRP exists in a pentameric form consisting of five monomers arranged in a donut-shaped structure [45]. The most widely used CRP detection methods are antibody-based immunoassays (IA) with very different dynamic ranges, from fg/L up to µg/L [46]. In contrast, eFBG-fibers were biofunctionalized with a CRP-specific, single-stranded DNA aptamer. Aptamers have been extensively studied as recognition elements in biosensors, since they possess several advantages compared to antibodies [47,48,49]. Aptamers can be selected in vitro in the so-called SELEX approach for a large variety of targets with high binding affinities, similar to antibodies. Then, they can be easily synthesized chemically and exhibit high stability at increased temperatures or large pH changes. Thus, aptamers are favorable recognition elements for diverse biosensing applications. To the end, we combined the advantages of aptamers and eFBGs for realizing a CRP detection system with excellent sensitivity.

## 2. Materials and Methods

### 2.1. Materials and Reagents

Single-mode optical fibers (SMF-28e) were purchased from Corning Optical Communications (Berlin, Germany). Ethanol, glycerol, (3-glycidyloxypropyl)trimethoxysilane (GOPTS), L-cysteine, phosphate buffered saline (PBS; pH = 7.4), H_2_O_2_ (ω = 30%), potassium hydroxide, and urea were purchased from Sigma-Aldrich (Taufkirchen, Germany). Acetic acid, CaCl_2_·2H_2_O, 1 M HCl, MgCl_2_·6H_2_O, L-ascorbic acid, KCl, NaCl, 1 M NaOH, H_2_SO_4_ (ω = 96%) and Tris base were purchased from Carl-Roth (Karlsruhe, Germany). Hydrofluoric acid (HF; ω = 40%) and immersion oil were purchased from AppliChem (Darmstadt, Germany). Water was purified with a Milli-Q purification system. Human recombinant C-reactive protein (CRP; β = 1 mg/mL) was purchased from BioCat (Heidelberg, Germany) and pooled human >97% CRP deficient plasma was purchased from Dunn Labortechnik (Asbach, Germany). The CRP-specific single-stranded DNA aptamer (CRP-40-17-3′SH) with a thiol group at the 3′-end was synthesized and HPLC was purified by Metabion (Planegg, Germany) and delivered at 100 µM in bidest. water. The sequence of CRP-40-17-3′SH was: 5′-CCC CCG CGG GTC GGC TTG CCG TTC CGT TCG GCG CTT CCC CTT TTT TTT T-C6-SH-3′ [50].

### 2.2. Writing of FBGs

**Figure 1:**
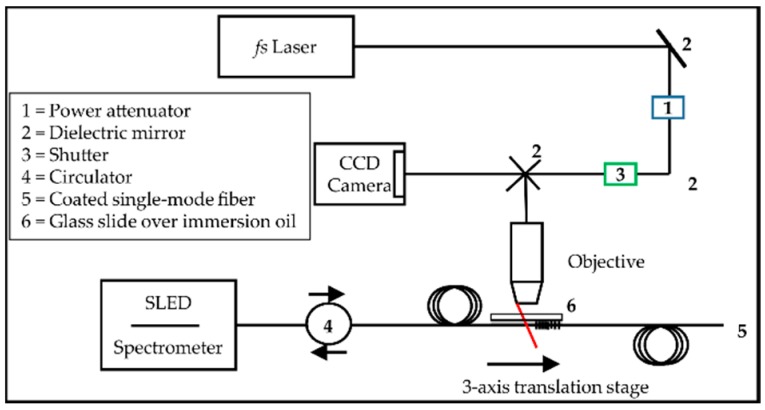
Experimental setup for fiber Bragg gratings (FBG) writing using ultrafast-laser-inscription, according to [23].

The experimental setup for the inscription process is shown in Figure 1. The used laser system consisted of commercially available Mai Tai seed laser and a Spitfire ACE amplifier (Newport Spectra-Physics, Darmstadt, Germany). The laser produced pulses at *τ*_p_ = 40 fs at a wavelength of *λ*_p_ = 800 nm, with an output power of *P*_av_ = 10 W and a repetition rate set to *f*_rep_ = 100 Hz. The laser beam was focused into the fiber core of the single-mode optical fiber without stripping the coating by using a microscope objective (LDA-Plan, 40× air objective, NA = 0.55). The laser pulse energy was adjusted to *E*_p_ ≈ 1 µJ at the objective back aperture. The optical fiber was mounted on a high precision 3-axis translation stage (XMS50; Newport, Darmstadt, Germany; ±0.75 µm on-axis accuracy) to guarantee an accurate positioning during the inscription process, while the fiber position was simultaneously monitored by a CCD camera (DMK 72AUC02, The Image Source, Bremen, Germany). This setup also allowed for bright-field image acquisition. In addition, the fiber was immersed in an oil film (Immersion oil for microscopy, fluorescence-free, Darmstadt, Germany; *n* = 1.480–1.482) and covered by a glass slide, in order to rectify the surface geometry presented to the incoming laser beam and thus to avoid a cylindrical lens effect from the surface curvature of the fiber during the inscription process [51]. An optical beam shutter (SH05; Thorlabs, Munich, Germany) was used to control the laser irradiation time. The inscription process was performed with a constant movement of the fiber on the translation stage and was stopped after achieving the desired grating length of 2.7 mm or a desired reflectivity of ~80%, respectively. The translation speed during inscription was set to *v* = 0.1058–0.1082 mm/s resulting in Bragg wavelengths *λ*_B_ = 1530–1565 nm. A fiber-coupled broadband superluminescent diode (SLED; Miopas, Goslar, Germany) with a central emission wavelength at 1550 nm and a 5 dB bandwidth of approximately 50 nm together with an integrated spectrometer with a resolution of Δλ = 0.18 nm was connected to the processed fiber, in order to measure the reflection spectrum online during the inscription process.

FBGs fabricated by PBP technique could be affected by stitching errors and losses induced by asymmetry of the grating structure inside the fiber core. Thus, the quality of the written gratings was checked randomly (*N* = 20 gratings from different days over a writing period of 11 months) by analyzing the grating homogeneity with light microscopic imaging. For this, parts of the gratings were imaged *via* bright-field illumination. Afterwards, grating homogeneity could be easily checked by analyzing the intensity profiles along the grating structures from the recorded images. Thus, the profile fitting with a simple sine function provided the period of the angle function, for which small fitting errors and similarity to the assumed grating period Λ_FBG_ would indicate minor influence of the above mentioned effects.

### 2.3. Signal Processing System

**Figure 2:**
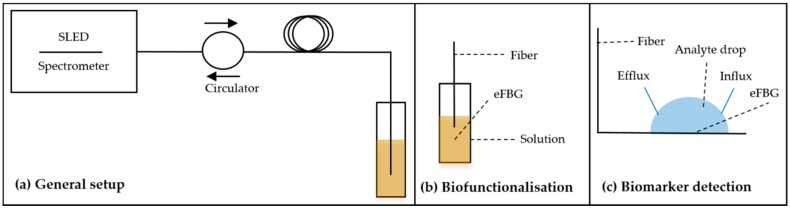
Overview of the used experimental setups. (**a**) General setup for signal processing; (**b**) Setup during bioassay formation; (**c**) Setup during analyte detection. For details, see text.

The experimental setup for all further biochemical treatments was partly the same as for the inscription process as shown in Figure 2. The SLED was connected to the processed fiber to measure the obtained reflectivity (Figure 2a). After dipping the FBG at the end of the fiber into the treatment solution, changes in *λ*_B_ and peak amplitude could be monitored in real time as well as single reflection spectra could be recorded (Figure 2). The biofunctionalization steps were performed in a vertical fiber position (Figure 2b), whereas the analyte detection was performed in a horizontal fiber position (Figure 2c). The biofunctionalization steps except for the etching procedure were performed in small reaction tubes under stirring at room temperature to guarantee a homogeneous solution during the whole incubation time and to minimize solution evaporation. The etching procedure was conducted in Falcon tubes due to the required larger volumes. For the analyte detection, the solution of the sample droplet being in contact with the FBG could be perfused using a peristaltic pump working at a flow rate of *v*_flow_ = 150 µL/min.

Due to an increasing mechanical fragility of the probe during the following fiber modification steps, fibers were mounted on a Teflon stick for all experiments starting from the etching procedure. This prevented fiber brakeage induced by mechanical stress and also minimized polarization dependent Δ*λ*_B_ changes, especially during analyte detection. Furthermore, the inscription of a second FBG, but with a different grating period and without etching, allowed for temperature control.

### 2.4. Fiber Etching Process

For removing the fiber cladding, the fiber was at first immersed in HF (ω = 40%) for 45 min. Afterwards, the fiber was cleaned with water, followed by a second etching step in a different HF solution (ω = 20%) until a certain value of Δ*λ*_B_~−1750 pm had been reached or the peak amplitude decreased below a threshold value of 20% of the initial value of the peak with the highest reflectivity. These empirical values guaranteed correct peak analysis when using a self-written python-based software tool and resulted in good sensitivities to SRI changes. Afterwards, the fiber was neutralized with 2 M KOH and subsequently rinsed with bidest. water. Then, the sensitivity of the resulting eFBG (*S*_eFBG_) was determined by immersing the fiber into solutions of different CaCl_2_ concentrations in the range of 0–40 wt%, corresponding to a linear increase of RI from *n* = 1.32–1.42 at an operating wavelength of *λ* = 1550 nm. The RI values were measured at different wavelengths in the range of *λ* = 403–938 nm by using a refractometer (Atago 1211 NAR-1T, Atago, Tokyo, Japan). Subsequently, RI values were extrapolated for the operating wavelength.

### 2.5. Biofunctionalization

A preceding two-step cleaning procedure was necessary, before the fiber could be biofunctionalized. At first, the fiber was immersed in piranha solution (H_2_SO_4_:H_2_O_2_ = 2:1, *v*/*v*) for 1 h. Then, the fiber was rinsed with bidest. water. Secondly, plasma cleaning occurred by exposing the fiber to low-pressure plasma (Zepto, Diener electronic, Ebhausen, Germany) for 5 min. After these cleaning steps, the fiber was immersed in 2 mL of a freshly prepared 2.5 vol% GOPTS solution for silanization. For this, GOPTS was dissolved in ethanol: H_2_O (95:5, *v*/*v*), which was set to pH = 4.9 by adding acetic acid. Silanization occurred overnight at room temperature in the dark and subsequently the fiber was rinsed three times in ethanol/water. Then, the fiber was treated with 1 µM aptamer dissolved in water, which was set to pH = 8–9 by adding 1 M NaOH, under constant stirring at room temperature in the dark overnight. Finally, the fiber was rinsed with water. GOPTS molecules, which did not react during the aptamer binding step, were blocked by fiber immersion into a L-cysteine solution (c = 50 mM in PBS, pH = 8.0) at room temperature for 2 h, followed by an additional washing step with the solvent to remove free L-cysteine. For correct three-dimensional aptamer folding, the aptamer-coated fiber was heated to 90 °C for 3 min and immediately chilled on ice for at least 5 min. Aptamer-coated eFBG fibers were stored at room temperature up to 3 h before starting a CRP recording series.

### 2.6. CRP Detection

The detection of CRP was evaluated by immersing the aptamer-coated eFBG fibers into solutions of different CRP concentrations in the range of 10^−11^–10^−3^ mg/L and simultaneous λ_B_ acquisition. For this, CRP has been dissolved in a modified aptamer buffer (140 mM NaCl, 5 mM KCl, 1 mM MgCl_2_, 1 mM CaCl_2_, 50 wt% glycerol, 20 mM Tris, pH = 7.4) [52]. The fiber was incubated for 10 min with a CRP solution and was then cleaned with 500 µL aptamer buffer. Due to the application of a perfusion setup, the fiber was not moved during the measurement of a CRP concentration series. Thus, disturbing effects during *λ*_B_ recordings as a result of fiber bending could be minimized. Finally, control experiments were performed to validate the eFBG fiber-optical biosensor. At first, fibers were biofunctionalized as described above, but without coupling the CRP-specific aptamer. Furthermore, CRP detection experiments were performed in the presence of interfering substances, in order to test the selectivity of the developed CRP-bioassay. On the one hand, this could be realized by adding two interfering substances at constant concentrations (1.8 mg/mL ascorbic acid and 1.8 mg/mL urea) into the modified aptamer buffer. One the other hand, >97% CRP deficient human plasma with a bunch of interfering substances could be used. Here, plasma was diluted with modified aptamer buffer to the lowest measureable CRP concentration assuming a residual plasma CRP content of <0.6 mg/mL (<3% of normal level with 20 mg/L) and was then used as constant background level for the CRP concentration series. However, the general experimental conditions and the used CRP concentration range in the control experiments were identical to that of the initial CRP detection experiments.

### 2.7. Data Analysis

For each Δ*λ*_B_ measurement, 10 reflection spectra were consecutively recorded (one per second), of which an average spectrum was calculated. All average reflection spectra were analyzed with a self-written python-based program. Thus, reflection peaks could be at first recognized and subsequently center wavelengths (Δ*λ*_B_), as well as peak amplitudes, were extracted from Gaussian fits. This resulted in a fit accuracy of Δ*λ*_fit_ = 5 pm, which can be understand as the maximum theoretical resolution achievable with the sensor according to the experimental and instrumental setup. Mean values and standard error of the mean (SEM) were calculated from several replicates. Graphical illustration was performed with OriginPro 2017 (OriginLab Corporation, Northampton, MA, USA).

## 3. Results and Discussion

### 3.1. Writing of FBGs

**Figure 3:**
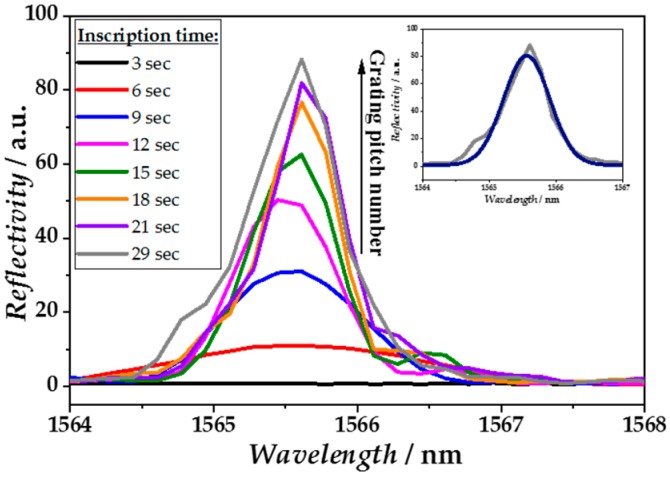
Reflection spectra of *λ*_B_ with increasing intensities indicating higher grating reflectivity; measured at different time points during the point-by-point inscription process. Inset: Recorded spectrum for 29 s, together with Gaussian fit.

The FBG fabrication method is important for obtaining gratings with best physical and optical properties. Here, the gratings were fabricated PBP via a nonlinear absorption process of a highly focused *fs*-pulsed laser leading to photoionization and permanent structural changes in the fiber core. Thus, RI was locally increased due to forming of a plasma in the laser focus [53,54]. The increase in the peak intensity of the reflected Bragg resonance wavelength *λ*_B_ during the PBP inscription process is shown exemplary in Figure 3, indicating an increasing grating reflectivity due to the increasing number of grating pitches.

**Figure 4:**
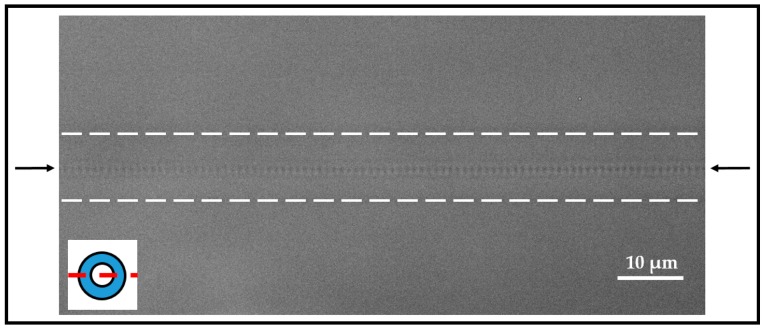
Microscopic image of part of a point-by-point written FBG. The black arrows indicate the position of the individual grating pitches. The dashed lines illustrate the core-cladding-interface. The inset indicates the optical section plane.

After an inscription time of 29 s, the sharp reflection peak exhibited a full width half maximum (FWHM) of <1 nm. In this work, gratings consisted of larger FWHM values (FWHM = 0.81 ± 0.01 nm, *N* = 345) in comparison to previously reported *fs*-written gratings (FWHM = 0.1–0.55 nm) [19,27]. This deviation could be mainly the result of different experimental setups such as average laser power or numerical aperture of the used objective. Furthermore, FWHM is dependent on the laser pulse energy, which is one factor of the resulting size of the *n*-modification shape [55]. However, high sensor sensitivity would be mainly the result of accurate detection of peak position changes. A microscopic view of part of a FBG written by the PBP method is shown in Figure 4, illustrating the point-like structure of RI changes in the fiber core in contrast to the RI change across the whole fiber cross-section due to an interference pattern when using the phase mask method. Thus, the optical fiber used for PBP inscription of FBGs requires no pre-treatment or prior preparation steps, but results in nicely arranged gratings exclusively within the fiber core. However, one requirement is a high precision in the fiber translation speed during the inscription process, which takes several tens of seconds per grating. Otherwise, the grating period will be irregular or the grating pitches will be arranged in different distances relative to the core-cladding interface, leading to rather poor reflection peaks and inconsistent SRI sensitivities.

**Figure 5:**
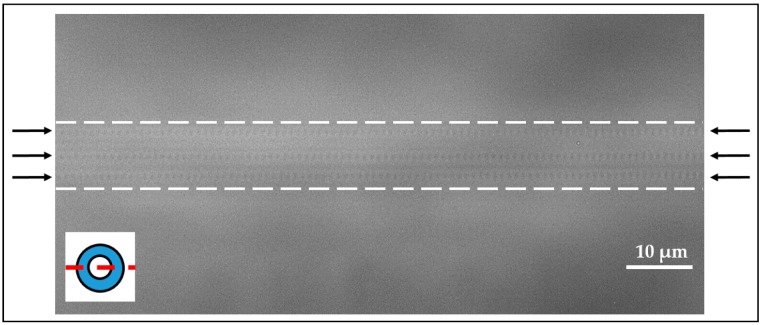
Microscopic image of part of a fiber including three FBGs in the fiber core next to each other. The gratings consisted of different pitch distances (different grating periods), so that all FBGs can be observed simultaneously. The black arrows indicate the positions of the individual gratings. The dashed lines illustrate the core-cladding-interface. The inset indicates the optical section plane.

Due to its advantages, the recent setup for ultrafast-laser-inscription can be also used to write more than one grating into the fiber core segment, just by changing the focal plane (Figure 5). By doing so, *λ*_B_ can be changed between the gratings by varying the translation speed, so that all written gratings can be monitored simultaneously within one reflection spectrum. This grating design could be then used to increase the overall sensor performance, since more gratings at the same fiber segment allow for compensating cross-sensitivities. However, this grating design has to be tested in further studies. In the following, only optical fibers with 2 FBGs written in the same focal plane, but spatially separated along the longitudinal axis were used. So, one grating could be biofunctionalized and the other one could be used as reference.

**Figure 6:**
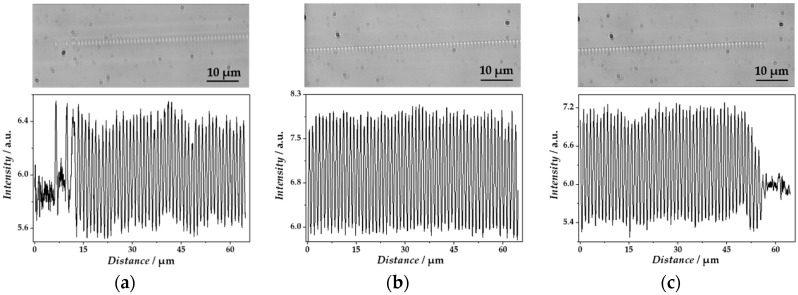
Microscopic images of a point-by-point written grating in the fiber core together with the corresponding intensity profile plots along the longitudinal fiber axis. (**a**) The starting region of the grating. (**b**) A middle-section of the grating. (**c**) The end-section of the grating.

Analysis of the light microscopic images of the written gratings unraveled homogeneously distributed grating pitches over the whole grating range (Figure 6) along the longitudinal fiber axis. A slight inhomogeneous pitch distribution could be only observed in the starting region of the grating (Figure 6a), whereas the middle- and end-sections were homogeneous (Figure 6b,c). The sine function fits of the intensity profiles along the longitudinal fiber axis resulted in periods of 528.2 nm up to 540.3 nm, with a standard error average of 0.2 nm (*N* = 20) for λ_B_ from 1530 nm up to 1565 nm, respectively. Here, the obtained periods corresponded to the half of the grating period (Λ_FBG_) and displayed a relative deviation from the theoretically expected grating periods of 0.2% (at *λ*_B_ = 1565 nm) and 0.1% (at *λ*_B_ = 1560 nm), respectively. Thus, the applied PBP technique resulted in highly homogenous gratings within the fiber core, leading to reproducible Bragg wavelength peaks of high reflectivity.

### 3.2. Fiber Etching Process

**Figure 7:**
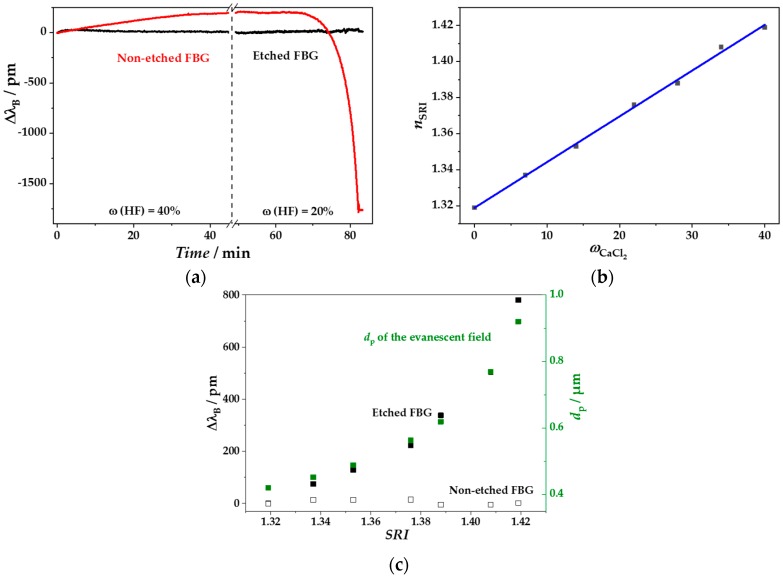
Fiber hydrofluoric acid (HF)-etching process. (**a**) Δ*λ*_B_ changes during the etching process of a FBG and of a FBG in a non-etching region as reference. (**b**) The corresponding curve between the mass concentrations of CaCl_2_ and their refractive index (RI) in water, calculated for λ = 1550 nm. (**c**) Peak maximum shift of the etched FBG (eFBG, black) and non-etched FBG (white) after the etching procedure in relation to surrounding refractive index (SRI) adjusted by different CaCl_2_ solutions at a wavelength of λ = 1550 nm. The calculated values of the corresponding penetration depth within the RI range (green) are calculated according to Equation (4).

For removing the cladding from the FBG-inscribed fibers, HF etching was performed. The etching process had to be monitored online, in order to stop the process immediately after reaching the appropriate fiber diameter without etching off the fiber totally. The online monitoring of Δ*λ*_B_ is exemplary shown in Figure 7a. The shift to higher wavelengths (Δ*λ*_B_ > 0 pm) in the first 45 min is mainly induced by the thermal expansion due to the exothermic reaction of HF and silica of the fiber. On the other hand, the subsequent shift to shorter wavelengths starting after approximately 65 min is mainly the result of changed *n*_eff_ with a higher influence of SRI of the medium during the ongoing cladding removal (Figure 7a) [56].

The etching procedure was stopped at a shift of approx. Δ*λ*_B_ = −1750 pm. Afterwards, fibers were immersed in CaCl_2_ solutions of different RI (Figure 7b) to determine the sensitivity *S*_eFBG_ of the resulting eFBGs according to:

(3) $$S_{\mathrm{eFBG}}=\frac{\Delta \lambda_{B}}{\Delta RI}$$

*S*_eFBG_ values up to 8 nm/RIU were observed for different batches of etched fibers. The *S*_eFBG_ value of the eFBG shown in Figure 7a,c was determined to 7.81 nm/RIU. Fiber with insufficient sensitivities <3 nm/RIU were rejected. As known from literature [8], the slope of Δ*λ*_B_ increased with higher RI (Figure 7c) and for simplicity an average sensitivity was calculated from the difference between the highest and lowest measured values. The reason for this non-linear behavior can be seen in the changed penetration depth (*d*_p_) of the evanescent wave according to [57]:

(4) $$d_{P}=\frac{\lambda }{2\pi \sqrt{n_{\mathrm{co}}^{2}\sin^{2}\theta -n_{\mathrm{cl}}^{2}}}$$

In Equation (4), *n*_co_ and *n*_cl_ are the RI values of fiber core and cladding, respectively, and *θ* is the incidence angle of the light at the core-cladding interface. In case of an eFBG, *n*_cl_ can be set to SRI, since such a fiber has been etched up to the core-cladding interface. The penetration depth is the distance at which the intensity of the evanescent wave is decreased to 1/e of its initial value at the core-cladding interface [58]. The RI values of the fiber core and cladding could be calculated by using the Sellmeier formula for fused silica [59,60] and the fractional refractive index change of the used fiber type Δ = 0.36% [61]. For a wavelength of λ = 1550 nm, this resulted in *n*_co_ = 1.4492 and *n*_cl_ = 1.4440, respectively. According to the Snell’s law at the core-cladding interface:

(5) $$\theta_{c}=\arcsin\left(\frac{n_{\mathrm{cl}}}{n_{\mathrm{co}}}\right)$$

The incidence angle (*θ*) has to be greater than the critical angle (*θ_c_*) for total internal reflection. The critical angle for a non-etched fiber was determined to *θ*_c_ = 85.1°. The critical angles in the shown RI range (Figure 7c) increased from *θ_c_* = 65.5–78.3°, therefore we assumed an incidence angle of *θ_c_* = 85.2° for all tested CaCl_2_ solutions. Thus, the penetration depth increased non-linearly from *d*_p_ = 0.4–0.9 µm, when increasing SRI from *n*_SRI_ = 1.32–1.42 (Figure 7c). The maximum penetration depth is almost reached at *n*_co_ [8]. Thus, the SRI sensitivity can be in principal enhanced on the one hand by using thinner etched fibers, but then with the drawback of larger mechanical fragility making them unsuitable for more robust applications. On the other hand, the sensitivity could be improved by increasing the measuring regime of *n*_cl_ or rather SRI, for instance by coating the fiber with an nm-scale, high-RI film [62], or by adding high-RI substances into the sample solution. Here, the following biomarker measurements were performed at SRI of approximately *n* = 1.40 by adding glycerol as a high-RI substance. This procedure led to a penetration depth of *d*_p_ ≈ 0.6 µm, which was large enough for the gradual detection of the subsequent biofunctionalization steps.

### 3.3. Biofunctionalization

**Figure 8:**
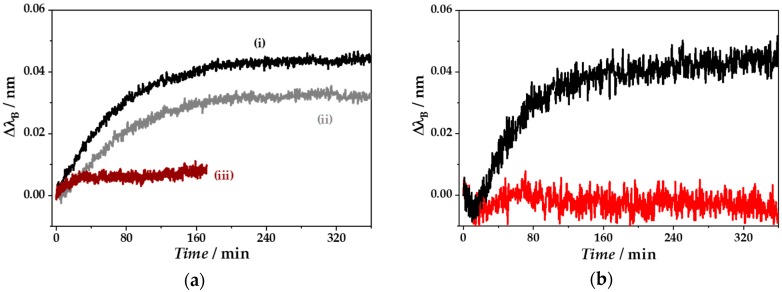
(**a**) Online monitoring of Δ*λ*_B_ during the fiber biofunctionalization steps including (i) silanization with (3-glycidyloxypropyl)trimethoxysilane (GOPTS) (black), (ii) coupling of the C-reactive protein (CRP)-specific single-stranded DNA aptamer as recognition element (grey), and (iii) blocking treatment with L-cysteine (brown). (**b**) Online monitoring of Δ*λ*_B_ during silanization with GOPTS of an etched (black) and non-etched grating (red) within the same fiber.

For specific biomarker detection, the etched and thus, SRI sensitized fibers had to be biofunctionalized. Here, each bioassay formation step could be monitored as shown in Figure 8. The plots in Figure 8a show exemplarily the bioassay formation of one fiber, in order to visualize the whole process from the etching process up to the analyte quantification. The temporal change of *λ*_B_ due to fiber immersion in GOPTS is shown in trace (i). The silanization results in Δ*λ*_B_ of 45 pm. The highest shift could be observed in the first 80 min. A plateau with a constant value of *λ*_B_ could be seen after 200 min, indicating the end of the reaction with a formed GOPTS layer. A statement about the layer uniformity was not possible. However, the possible formation of a second GOPTS layer could be excluded due to the acidic experimental conditions and the pH-dependence of the reaction [63]. The shift of *λ*_B_ by immersion of the fiber with the CRP-specific aptamer as recognition element is shown in trace (ii). This reaction showed principally the same trend as seen for the immersion in GOPTS solution. A constant Bragg wavelength shift could be observed with Δ*λ*_B_ of approximately 30 pm after 180 min, although the strongest effect could be already recorded in the first 120 min. The increase of *λ*_B_ during the blocking step with L-cysteine accounted for approximately 6 pm within 30 min. After that, a constant peak position could be observed as shown in trace (iii). L-cysteine was chosen because of its relatively small size (121 Da) and its property to react with the functional thiol or amino groups dependent on the solvent´s pH [63].

Furthermore, the second, non-etched grating of each fiber was used to control temperature-induced SRI effects. The example in Figure 8b showed only a slight temperature effect in the initial 10 min, indicating a negative change in Δ*λ*_B_ (red curve). Afterwards, Δ*λ*_B_ changes of the non-etched grating were within the range of Δ*λ*_fit_ accuracy. Thus, Δ*λ*_B_ changes due to biofunctionalization steps of the etched grating (black curve) were significantly different from just temperature effects. In addition, no further significant temperature effects could be observed at following biofunctionalization steps or during the analyte detection procedure.

**Figure 9:**
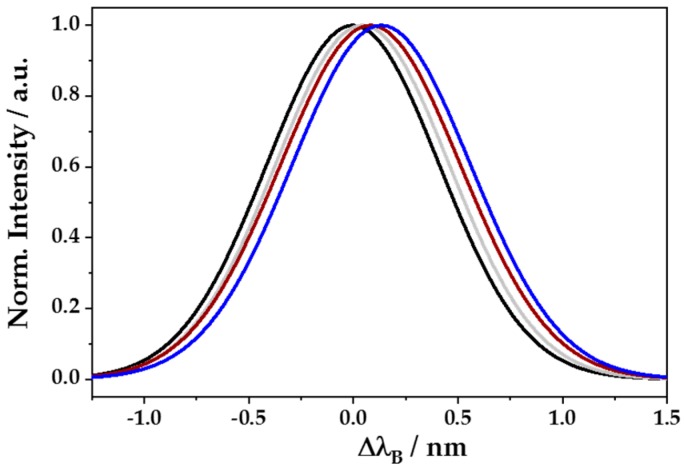
Gaussian fits of reflection spectra with Δ*λ*_B_ shifted to larger values at the end of each fiber biofunctionalization step, including the initial untreated eFBG (black), silanization with GOPTS (grey), coupling of the CRP-specific single-stranded DNA aptamer together with the blocking treatment with L-cysteine for visibility (brown), and analyte binding to 8.7 × 10^−12^ M CRP (blue). Changes are shown relative to the initial untreated eFBG (black, Δ*λ*_B_ = 0) and the final Δ*λ*_B_ of each step is the starting point for the subsequent step.

Finally, after silanization, aptamer binding, and blocking step, biofunctionalized eFBG fibers could be used for testing their sensitivity to the biomarker CRP. An example of recorded reflection spectra of such a procedure is shown as Gaussian fits due to clarity in Figure 9. Each step led to higher wavelengths (Δ*λ*_B_ > 0). The total shift accounted for Δ*λ*_B_ = 137 pm, including silanization (45 pm), aptamer binding (33 pm), blocking step (5 pm), and CRP binding (54 pm). In addition, no significant changes of the peak shape and FWHM (0.97–1.02 nm) could be observed, even though the analysis of peak amplitude changes was sufficient for CRP detection.

### 3.4. CRP Detection

**Figure 10:**
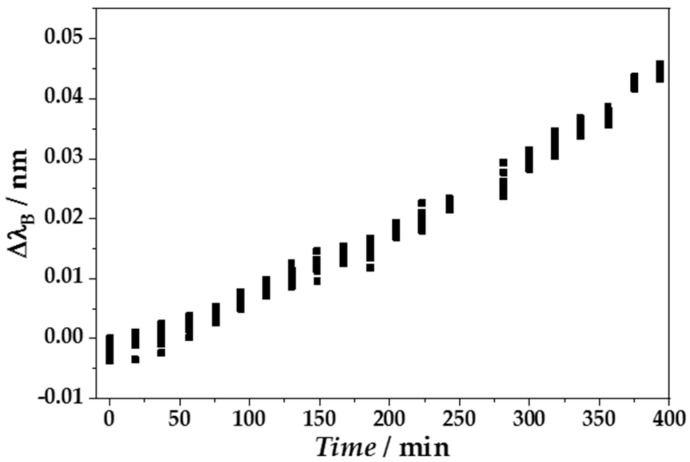
Online monitoring of Δ*λ*_B_ of one representative CRP-sensitive eFBG fiber during the application of a CRP concentration series (7.8 × 10^−19^–8.7 × 10^−12^ M) in 20 concentration steps over a time period of 375 min. For each CRP concentration, 10 reflection spectra were recorded subsequently (black dots).

The application of CRP to a biofunctionalized eFBG fiber resulted in a continuous increase in Δ*λ*_B_ to more positive values Δ*λ*_B_ > 0 pm with increasing CRP concentration over time from 7.83 × 10^−19^ M to 8.70 × 10^−12^ M (Figure 10). The single data points in Figure 10 indicate the variance of the Bragg wavelength fitting procedure for 10 subsequently recorded reflection spectra for each CRP concentration, also including small vibrations during the single measurements. Because of this, the fit accuracy was set to Δ*λ*_fit_ = 5 pm. The increase in Δ*λ*_B_ indicated a continuous adsorption of CRP molecules to the aptamer-coated fiber surface, leading to higher SRI nearby the eFBG and thus an increasing *n*_eff_. The Bragg wavelength in the presence of buffer without CRP was set to zero, in order to eliminate background effects.

The CRP binding to the aptamer-coated fiber surface can be explained with the Langmuir-Freundlich adsorption isotherm assuming either homogeneous CRP binding to identical and independent binding sites or heterogeneous CRP binding to binding sites in the case of cooperativeness. Finally, one would assume a monolayer coverage because of the relatively small size of the aptamer in contrast to the size of the pentameric structured CRP molecule. The Langmuir-Freundlich isotherm combines the behavior of Langmuir and Freundlich isotherms for a homogeneous and a heterogeneous monolayer formation, respectively. With the assumption that Δ*λ*_B_ is proportional to the adsorption process this can be analyzed according to [64,65]:

(6) $$\Delta \lambda_{B}=\Delta \lambda_{B,\max}\left[\frac{{\left(Kc_{\mathrm{CRP}}\right)}^{\beta }}{1+{\left(Kc_{\mathrm{CRP}}\right)}^{\beta }}\right]$$

where Δ*λ*_B_,_max_ is the maximum Bragg wavelength shift due to occupation of all CRP-binding sites, *K* is the equilibrium binding constant, *c*_CRP_ is the molar CRP concentration (1 mg/L = 8.7 nM by using a molar mass of *M* = 115,000 g/mol), and *β* is the empirical Langmuir-Freundlich parameter being between 0–1 and indicating the degree of binding heterogeneity. If *β* < 1, when a heterogeneous system can be assumed, such as binding of the first analyte molecule would somehow influence the binding of a second molecule. In the case of a homogeneous system, *β* = 1, Equation (6) results in the Langmuir adsorption isotherm. In the case of very small analyte concentrations or a very small equilibrium constant (*c*_CRP_ → 0 or *K* → 0), Equation (6) would result in the Freundlich isotherm:

(7) $$\Delta \lambda_{B}=K^{\prime }c_{\mathrm{CRP}}^{\beta }$$

**Figure 11:**
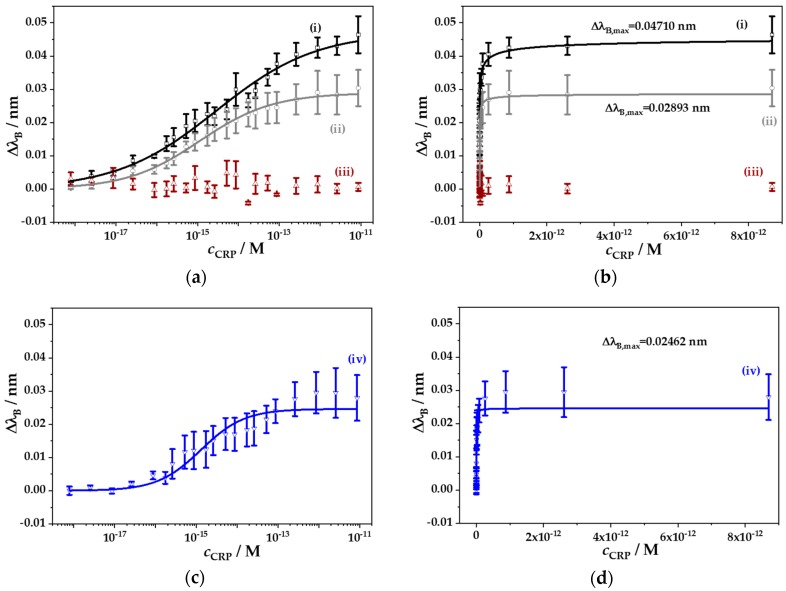
The Bragg wavelength shift Δ*λ*_B_ of biofunctionalized eFBG fibers as a function of a CRP concentration range (i) without any interfering substances (black), (ii) in presence of the interfering substances urea (1.8 mg/mL) and ascorbic acid (1.8 mg/mL) (grey), (iii) without fiber coupling of the CRP-specific aptamer and any interfering substance (brown), and (iv) in presence of diluted CRP deficient human plasma (blue). Data were fitted to the Langmuir-Freundlich isotherm according to Equation (6) (solid lines), with fit qualities of (i) R^2^ = 0.988, (ii) R^2^ = 0.989, and (iv) R^2^ = 0.951, respectively. Data of Δ*λ*_B_ vs. c_CRP_ are shown in a semi-logarithmic plot (**a**,**c**), as well as in a linear plot (**b**,**d**) to indicate the asymptotic characteristic of Δ*λ*_B_ within the CRP concentration range. Means ± SEM for (i) N = 5, (ii) N = 8, (iii) N = 3, and (iv) N = 3.

Within the tested CRP concentration range, Δ*λ*_B_ showed a sigmoidal growth with increasing CRP concentration in a semi-logarithmic plot, as expected from theory (Figure 11a). The nonlinear regression analysis of the CRP recording series using the Langmuir-Freundlich isotherm model resulted in a maximum shift Δ*λ*_B_,_max_ = (47.1 ± 2.3) pm and a binding constant *K* = (3.48 ± 1.28) × 10^5^ nM^−1^, with an acceptable goodness of fit being R^2^ = 0.988 (Figure 11b). The parameter of heterogeneity was calculated to be *β* = 0.355 ± 0.022.

From this, further biosensor parameters could be determined [37,66,67,68]. At first, the theoretical surface density concentration *σ*_max_, indicating the CRP concentration when all aptamer binding sites are occupied, can be calculated. Here, one assumes a monolayer coverage of the fiber surface and an average length of a CRP pentameric molecule of *d* = 11.13 nm [45] leading to:

(8) $$\sigma_{\max}=\frac{M}{N_{A}}\frac{1}{d^{2}}$$

where *M* = 115,000 g/mol is the estimated molecular weight of a CRP pentameric molecule given by the manufacturer and *N*_A_ = 6.02 × 10^23^ mol^−1^ is the Avogadro constant. Thus, the surface density concentration at CRP saturation of *σ*_max_ = 1.542 ng/mm^2^ was constant for each reported experiment. The sensitivity of the CRP-specific biosensor *S*_BS_ when using CRP-40-17-SH-3′ as recognition element is then given by:

(9) $$S_{\mathrm{BS}}=\frac{\Delta \lambda_{B,\max}}{\sigma_{\max}}$$

resulting in a sensitivity of *S*_BS_ = 0.031 nm/(ng/mm^2^). The theoretical detection limit of the eFBG biosensor *DL*_BS_ could be determined according to:

(10) $$DL_{\mathrm{BS}}=\frac{\Delta \lambda_{\mathrm{fit}}}{S_{\mathrm{BS}}}$$

Assuming a fit accuracy of Δ*λ*_fit_ = 5 pm when performing Gaussian fits to the Bragg wavelength peaks, the theoretical limit of detection of *DL*_BS_ = 0.164 ng/mm^2^ could be calculated. Because of this relationship, the theoretical detection limit of the shown eFBG biosensor would be predominately determined by the resolution of the spectrometer or rather the fitting quality in our measurements. FBGs with higher reflectivity will result in better signal to noise ratios (SNR), allowing lower theoretical limits of detection. Therefore, by using the Langmuir-Freundlich isotherm Equation (6) and the instrumental fit accuracy, one can evaluate the CRP concentration corresponding to a Bragg wavelength shift equal to the fitting accuracy, according to:

(11) $$c_{\mathrm{CRP},\lim}=\frac{1}{K}{\left(\frac{\Delta \lambda_{\mathrm{fit}}}{\Delta \lambda_{B,\max}-\Delta \lambda_{\mathrm{fit}}}\right)}^{\frac{1}{\beta }}$$

The CRP concentration limit of *c*_CRP,lim_ = 7.1 × 10^−18^ M (≡0.82 pg/L) would lead to a Bragg wavelength shift of Δ*λ*_B_ = 5 pm, the minimal detectable shift with the present setup. According to [69], the value of *c*_CRP,lim_ can be understood as the minimum detectable concentration that the sensor is able to reliably detect.

To evaluate the specificity of CRP binding to the eFBG biosensor, control experiments were performed in the presence of interfering compounds usually available in blood serum. Thus, the CRP concentration was varied between 7.83 × 10^−19^ M and 8.70 × 10^−12^ M, whereby maintaining constant concentrations of ascorbic acid and urea, each with 1.8 mg/mL. Again, the tested CRP concentration range resulted in a sigmoidal change of Δ*λ*_B_ (Figure 11a), which could be successfully fitted to the Langmuir-Freundlich isotherm (Figure 11b). However, due to the presence of the interfering substances, the maximum shift of Δ*λ*_B_,_max_ = (28.9 ± 1.1) pm was reduced by 38%. In addition, the binding constant increased to *K* = (8.88 ± 2.22) × 10^5^ nM^−1^, with a parameter of heterogeneity being *β* = (0.486 ± 0.030). Thus, the biosensor sensitivity decreased slightly to *S*_BS_ = 0.019 nm/(ng/mm^2^) and the theoretical detection limit increased to *DL*_BS_ = 0.267 ng/mm^2^. Thus, the minimal detectable CRP concentration increased by almost one order of magnitude to *c*_CRP,lim_ = 4.5 × 10^−17^ M (≡5.17 pg/L).

In order to check a broader range of interfering substances, CRP deficient human plasma was diluted to the CRP concentration detection limit and was then used as buffer, in which the CRP concentration series could be tested. The influence of plasma substances resulted in a further decrease of Δ*λ*_B_,_max_ = (24.6 ± 1.8) pm (Figure 11c,d). The binding constant increased to *K* = (7.68 ± 2.76) × 10^5^ nM^−1^, similar to the system with two interfering substances, but the parameter of heterogeneity increased to a value of *β* = (0.816 ± 0.105). As expected, the biosensor sensitivity [*S*_BS_ = 0.016 nm/(ng/mm^2^)] and the theoretical detection limit [*DL*_BS_ = 0.313 ng/mm^2^] further declined, and the minimal detectable CRP concentration increased to *c*_CRP,lim_ = 2.4 × 10^−16^ M (≡27.6 pg/L). This result indicates that low CRP concentrations of normal human serum (*c*_CRP_ < 10 mg/L) [70] could be easily detected, even in the presence of interfering substances in human serum [71]. However, without coupling of the CRP-specific aptamer to the eFBG fiber, no significant changes in Δ*λ*_B_ upon CRP addition could be observed (Figure 11a), indicating negligible unspecific CRP binding on the fiber surface.

For performance evaluation of the new CRP-sensitive eFBG fiber-optical biosensor, selected literature-known, CRP-specific optical biosensors, as well as fiber-optical biosensors, for different analyte detection are listed in Table 1. Thus, detectable concentration limits of CRP-sensitive optical biosensors are in the low pg/L up to mid µg/L range, depending on the used biosensor design. However, our developed biosensor showed a low limit of 0.8–27.6 pg/L. The only reported CRP-sensitive biosensor design comparable to that of this study exhibited a five orders of magnitude higher detectable concentration limit with another dynamic range of 10 µg/L to 100 mg/L [72]. The different performances of both eFBG-based biosensors might be the result of different fabrication strategies concerning the FBG writing process. In addition, the fiber immobilization of aptamers in contrast to larger antibodies might be also advantageous in detecting low CRP concentrations. When comparing with other fiber-optical biosensors for analytes comparable in size to CRP, the detection limit of the developed biosensor is similar to the reported ones being in the low pg/mm^2^ range, although it is almost one order of magnitude higher (Table 1). Indeed, a larger sensing surface area or higher sensor porosity by immobilizing nanoparticles on the fiber surface could lead to an optimized interaction between the evanescent field and the sensitive layer, thus yielding a higher sensor sensitivity [9].

## 4. Conclusions

Fiber-optical biosensors based on fiber gratings detecting tinny refractive index changes are a beneficial approach for label-free sensing with excellent performance in terms of sensitivity and detection limits. Depending on the used grating type (e.g., FBG, TFBG, LPG) and the recognition element (e.g., antibody, aptamer), different platforms have been developed. Here, we used the advantages of FBGs since they exhibit a simply analyzable signal in terms of a narrowband Bragg wavelength peak, therefore can be easily multiplexed, and they allow the detection in the reflection operation mode, therefore can be easily used in remote sensing. However, so far only UV phase mask-written FBG fibers are reported for biochemical sensing. Here, we showed for the first time PBP-inscription of FBGs by using an *fs*-pulsed laser for generating fiber-optical biosensors. Homogeneously distributed grating pitches resulted in reflection peaks of high intensity. In contrast to UV light-inscribed gratings, no fiber pre-treatment is necessary due to direct writing of single grating pitches within the fiber core. Therefore, this process is quite flexible and will definitely result in more advanced application features in future, also allowing for usage of different fiber types without setup changing [81]. For high SRI sensitivity, as needed for biochemical sensing, the fibers were etched. Indeed, higher sensitivities of eFBGs can be mainly achieved with larger and larger cladding etching. However, this comes along with the drawback of increased mechanical fragility, preventing their mobile application. The obtained sensitivity of the bare eFBG to SRI was ~8 nm/RIU, which is two to three orders of magnitude lower than earlier reported FOBs such as the plasmonic based sensors [82]. Nonetheless, the showed eFBG-based FOB in combination with aptamers as recognition element resulted in *c*_lim_ values, which were quite comparable to TFBG- and LPG-based FOBs (Table 1). The usage of fixed eFBG fibers, but in combination with peristaltic pumps, as shown here, or with microfluidic tools, can still lead to a quite flexible and adaptable biosensor platform. In contrast to even stronger cladding etching, the sensitivity of FBGs to SRI changes can be also enhanced by adjusting the medium RI to higher starting values, as performed here by adding glycerol [8]. Another approach for increasing the FBG sensitivity would be the immobilization of gold nanoparticles, since they induce RI dependent waveguide losses. Thus, the shift and the intensity of Bragg wavelength are SRI-sensitive [29]. Interestingly, bare FBG fibers without any etching but antibody immobilization at fiber surface have also been recently shown for successful *E. coli* detection. There, strain-induced Bragg wavelength changes of approximately 25 pm during bacteria binding could be observed [83]. With the new CRP-sensitive eFBG fiber-optical biosensor, we showed a quite low limit of detection of 0.8 pg/L, with a dynamic range up to ~1 µg/L. Although the CRP blood concentration is maintained in the range of 1–200 mg/L, this low detection level could still be beneficial. One advantage is the need of only very small sample volumes (in the low µl-range), since dilution by 6–9 orders of magnitudes will still result in detectable CRP concentration ranges. On the other hand, the low detection limit of this eFBG biosensing scheme could be possibly adapted to scenarios in which the detection of very low amounts of analytes is definitely required. One prominent example could be the detection of endotoxins in food or biomedical products, since their detection in the very low pg/L range is requested and underlies strict formalities [84,85]. However, based on the results presented here, the adaption to other analytes of different complexity will be part of further investigations.

## Figures and Tables

**Table 1:** List of selected literature-known C-reactive protein (CRP)-specific optical biosensors and other fiber-optical biosensors (FOB), together with their characteristic properties.

Type of Biosensor *	Detected Analyte	Detection Limit *DL*_BS_	Concentration Limit *c*_lim_ #	Ref.
FOB based on eFBG and immobilized CRP-specific aptamer	CRP	164–313 pg/mm^2^	0.8–27.6 pg/L	this work
FOB based on LMR and immobilized CRP-specific aptamer	CRP		62.5 µg/L	[73]
FOB based on eFBG and immobilized CRP-specific antibody	CRP		10 µg/L	[72]
FOB-based SPR-sensor and immobilized CRP-specific antibody	CRP		9 µg/L	[74]
SPRi-aptasensor based on immobilized CRP-specific aptamer	CRP		5 pg/L	[75]
Microfluidic chemiluminescent assay based on CRP-specific aptamer and antibody	CRP		12.5 µg/L	[76]
TIRFM-assay based on molecular switching fluorescence of FAI-PEA interaction	CRP		800 aM (92 pg/L)	[77]
FOB based on eFBG and coated APBA-RGO	D-glucose Hemoglobin HbA_1c_		1 nM (180 ng/L) 86 nM (1.5 mg/L)	[78]
FOB based on LPG and biotin-coated NP	Streptavidin	19 pg/mm^2^	195 µg/L	[37]
FOB based on eFBG and coated SWNT and dendrimer polymers	Concanavalin A		1 nM (110 µg/L)	[79]
FOB based on WEFT and immobilized IgG	IgG antibody	0.73 pg/mm^2^ 3.38 pg/mm^2^	0.2 nM (1.2 µg/L) 4.9 nM (31.6 µg/L)	[67]
FOB based on LPG and immobilized IgG	IgG antibody	5 pg/mm^2^		[80]
FOB based on tilted FBG and immobilized BSA	BSA antibody	12–13 pg/mm^2^	86–525 µg/L	[68]
Optical biosensor based on slot-waveguide microring resonator and coated BSA antibody/BSA	BSA BSA antibody	16 pg/mm^2^ 28 pg/mm^2^		[66]

* APBA-RGO aminophenylboronic acid functionalized reduced graphene oxide; BSA bovine serum albumin; FAI fluoresceinamine isomer 1; IgG immunoglobulin G; LMR lossy mode resonances; LPG long period grating; NP nanoparticles; PEA O-phosphorylethanolamine as CRP ligand; SPRi surface plasmon resonance imaging; SWNT single walled carbon nanotubes; TIRFM total internal reflection fluorescence microscopy; WEFT waist-enlarged fusion taper in a single-mode fiber. # Data in brackets was calculated from published molar concentrations using known molecular weights.
